# Tuning the Mesopore Structure of Polyethylene Glycol Terephthalate (PET)-Derived Hard Carbon for High-Capacity Sodium-Ion Batteries

**DOI:** 10.3390/ma18051166

**Published:** 2025-03-05

**Authors:** Chupeng Wang, Mingsheng Luo, Shiqi Song, Maochong Tang, Xiaoxia Wang, Hui Liu

**Affiliations:** 1 School of Materials Science and Engineering, East China University of Science and Technology, Shanghai 200237, China; chris20182171@163.com (C.W.); 15800650314@163.com (M.L.); 14747690106@163.com (S.S.); 17764062050@163.com (M.T.); 2 School of Mechanical and Power Engineering, East China University of Science and Technology, Shanghai 200237, China; 3 Zhuguangya Institute of Advanced Science and Technology, Shanghai 201306, China; huiliu0901@163.com

**Keywords:** hard carbon, sodium-ion batteries, PET plastic waste, mesopore

## Abstract

Hard carbon (HC) is considered to be a highly promising anode material for sodium-ion batteries. However, the synthesis conditions and pore structure regulation are still challenging for high-capacity sodium-ion storage. In this study, HCs using polyethylene glycol terephthalate (PET) as a carbon resource and ZnO as a nanopore template were synthesized and systematically investigated. By optimizing the additive amount of zinc gluconate, the starting material for ZnO, PET-derived HCs with a proper mesoporous structure were obtained. The as-prepared hard carbon demonstrated a high reversible capacity of 389.42 mAh·g^−1^ at 20 mA·g^−1^, with the plateau capacity accounting for 68%. After 75 cycles, the discharge capacity stabilized at 367.73 mAh·g^−1^ with a retention ratio of 89.4%. The rate performance test indicated that a proper mesopore structure helped to improve the sodium-ion diffusion coefficient, effectively enhancing the charge–storage kinetics. This work provides a promising strategy for converting PET into valuable carbon materials for application in the field of renewable energy technology.

## 1. Introduction

With the rapid development of renewable energy, low-cost and large-scale energy storage technology is urgently needed. Although lithium-ion batteries (LIBs) have been widely utilized in the field of energy storage and electric vehicles, limited lithium resources and increased demand hinder their further application. Alternatively, sodium-ion batteries (SIBs) are considered to be a promising substitute due to their high security and potential economic advantages. In recent years, SIBs have been successfully commercialized in the fields of energy storage and two-wheeled electric vehicles [1,2,3,4]. However, the most commonly used graphite anode in LIBs shows a low sodium storage capacity due to the high ionization potential energy of sodium ions [5,6]. Fortunately, hard carbon (HC), with highly disordered structures and expanded interlayer spacing, shows excellent sodium-ion storage characteristics and has been considered to be an ideal material for SIBs [7,8,9,10]. Two main kinds of precursors have been studied to prepare hard carbon, biomass and polymers. Generally, biomass precursors contain a lot of ash and impurities, which need to be removed before carbonization. Consequently, pre-treatments and post-treatments for biomass precursors are usually complicated. Moreover, different biomass precursors with specific structural features directly affect the morphology and structure of the obtained hard carbon, which makes studies on the structure–performance relationship more challenging [11,12,13]. Polymer resin is the other kind of precursor for hard carbon. Resin-based hard-carbon materials exhibit good performance for sodium storage. However, most polymer precursors are synthesized in complex ways using high-price organic compounds, leading to a high cost for the preparation of hard-carbon materials [14].

The storage mechanism of sodium in hard carbon has been widely investigated in recent years [15]. The charge–discharge curve of hard carbon is divided into the slope region (>0.1 V) and the plateau region (<0.1 V). Generally, the capacity of the slope region is attributed to the surface adsorption of sodium ions, and the capacity of the plateau region mainly originates from the intercalation layer and pore filling of the sodium ions (<0.1 V) [16,17,18,19]. Consequently, the sodium storage capacity is closely related to the microstructure of the hard carbon, such as the specific surface area, functional groups, pores, and defects. The layer spacing (d_002_) and microcrystal size (La and Lc) of graphite domains have been shown to affect the capacity of the plateau region. The carbon-layer spacing of hard carbon refers to the average distance between adjacent carbon atom layers in the local graphitic-like microregion in the hard carbon, which is an important parameter used to measure its structural order and performance. La represents the domain size within the carbon-layer plane (the graphene lamellar direction), reflecting the ordered region size of the carbon six-membered atomic ring network. Lc represents the stacking height of carbon layers along the vertical direction; that is, the average stacking-layer number of carbon layers in graphite-like microcrystals. The sodium storage capacity of hard carbon can be significantly increased by adjusting the appropriate d_002_, La, and Lc [15]. In addition, it has been reported that pore structure also has an important effect on capacity in the plateau region, especially a closed-pore structure [20,21,22]. In order to adjust the porous microstructure, template agents are usually used. However, the removal of template agents involves using a solution of acid or alkali. The synthesis process is commonly complicated and environmentally unfriendly. Therefore, it is of significant importance to develop a facile way to prepare hard carbon with proper porous structures [23,24].

At present, polyethylene glycol terephthalate (PET) is widely used in daily life, and millions of tons of waste plastics are produced every year, which causes extremely serious environmental and health issues [25,26]. Therefore, the conversion of waste PET into high-value-added products not only promotes sustainable development, but also plays an important role in protecting the environment. Recently, hard carbon pyrolyzed from PET was reported to have been used as an anode material for a SIB, which showed acceptable reversible capacity [27,28,29]. However, at present, the preparation of hard carbon using PET as the precursor has only been studied from the aspect of pyrolysis temperatures and heating conditions. Limited research on modifications to the morphology and microstructure of PET-derived hard carbon has been reported. Therefore, an optimized method should be explored to further improve the sodium storage performance of PET pyrolysis hard carbon.

In this work, we used PET as a precursor to prepare hard carbon with proper porous structures by introducing a small amount of zinc gluconate. The zinc gluconate decomposed during heat treatment with the formation of ZnO, which worked as a template agent. At high temperatures, ZnO was also reduced by C and the produced Zn vaporized because of its low vaporization temperature (907 °C), which led to the bulk-etching of the graphitic layers with the formation of pores. The optimized hard-carbon anode exhibited excellent electrochemical properties, with a high reversible capacity of 389.42 mAh·g^−1^ at a current density of 20 mA·g^−1^. The capacity did not significantly decline after 75 cycles, and the capacity retention ratio was 89.4%. Furthermore, the addition of zinc gluconate improved the diffusion ability of Na ions and reduced the charge transfer impedance. This work proposed an effective method to recycle PET and transform it into high-value-added materials through reasonable modifications.

## 2. Materials and Methods

### 2.1. The Synthesis of PET-Derived Hard Carbon

The PET-derived hard carbon was prepared by a simple one-step direct heating method using PET as the precursor. Firstly, waste PET beverage bottles (food grade, from Nongfu Spring Co., Hangzhou, China) were cut into small pieces with a side length of about 5 mm. The fragments were ultrasonically treated in anhydrous ethanol for 30 min, then cleaned with deionized water 3 times, and dried at 80 °C for 12 h. Furthermore, 3 g of the dried PET pieces was ground in an agate mortar for 20 min, then pyrolyzed in an argon stream at 600 °C for 30 min in a tube furnace, and then heated to 1400 °C for 30 min at a heating rate of 5 °C·min^−1^. The obtained materials were ground into powders after cooling and labeled as HC1400. In particular, zinc gluconate powders (purity 98%, from Macklin Co., Shanghai, China) were ground with PET pieces and the mixture was subjected to heat treatment in the same process. The obtained hard carbon was denoted as ZGHCx, where x represented the mass ratio of PET to zinc gluconate (x = 8, 16, 32, and 50). In particular, ZG1400 was obtained from the direct pyrolysis of zinc gluconate at 1400 °C.

### 2.2. Material Characterization Methods

The surface morphology of the samples was characterized using scanning electron microscopy (SEM; ZEISS Sigma 300, ZEISS, Oberkochen, Germany). The phase composition and microstructure of the samples were examined using X-ray diffraction (XRD; Ultima IV, Rigaku, Tokyo, Japan) with Cu-Kα radiation and transmission electron microscopy (TEM; FEI Talos F200X G2, Thermo Fisher Scientific, Waltham, MA, USA). Thermal gravimetric analyses (TGA; Netzsch STA-2500, Netzsch, Berlin, Germany) and Fourier transform infrared spectroscopy (FTIR; Thermo Fisher Scientific Nicolet iS20, Thermo Fisher Scientific, Waltham, MA, USA) were used together to detect the mass changes in the precursor and the gaseous products during the pyrolysis process in a nitrogen atmosphere from 30 °C to 800 °C. Surface element analyses of the samples were conducted using X-ray photoelectron spectroscopy (XPS; Thermo Fisher Nexsa, Thermo Fisher Scientific, Waltham, MA, USA). Raman spectroscopy (Thermo Scientific DXR, Thermo Fisher Scientific, Waltham, MA, USA) was characterized using a laser wavelength of 532 nm at wave numbers from 800 cm^−1^ to 2000 cm^−1^. In addition, the pore and specific surface areas were detected using N_2_ adsorption/desorption isotherms (Micromeritics ASAP 2460, Micromeritics, Norcross, GA, USA).

### 2.3. Electrochemical Measurements

CR2025 half coin cells were assembled using Na metal and glass fiber (Whatman, GF/C) as the counter electrode and separator, respectively. The obtained HCs were mixed with super-P and PVDF at a mass ratio of 8:1:1 in N-methyl-2-pyrrolidone (NMP) to form a homogeneous slurry, then the slurry was coated on a piece of Cu foil with a thickness of about 50 μm. After the wet slurry was dried at 120 °C for 12 h in a vacuum oven, an anode with a diameter of 12 mm was prepared. In addition, a mixture of 1 M NaPF_6_ dissolved in ethyl carbonate (EC), diethyl carbonate (DEC), and ethyl methyl carbonate (EMC) (volume ratio of 1:1:1) was used as the electrolyte. The cells were assembled in an argon-filled glove box, where the O_2_ and H_2_O concentrations were both controlled to lower than 0.01 ppm. The charge and discharge tests were carried out using a Neware battery cycler (CT-4008Tn-5V10mA, Neware, Shenzhen, China) between 0 V and 2.5 V at 30 °C. The galvanostatic intermittent titration technique (GITT) parameter was set with a current density of 20 mA·g^−1^, a current pulse duration of 0.5 h, and an interval of 2 h. Cyclic voltammetry (CV) and electrochemical impedance spectroscopy (EIS) were tested using a CHI660E workstation (Chen Hua, Shanghai, China).

## 3. Results

### 3.1. Characterization of Materials

**Figure 1:**
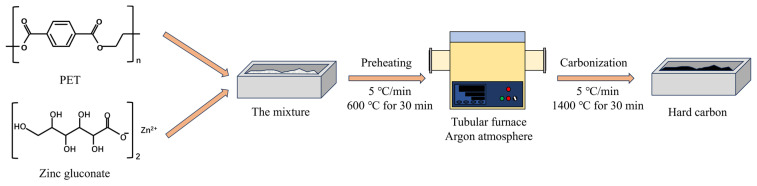
Schematic diagram of hard-carbon preparation using co-pyrolysis of PET and zinc gluconate.

**Figure 2:**
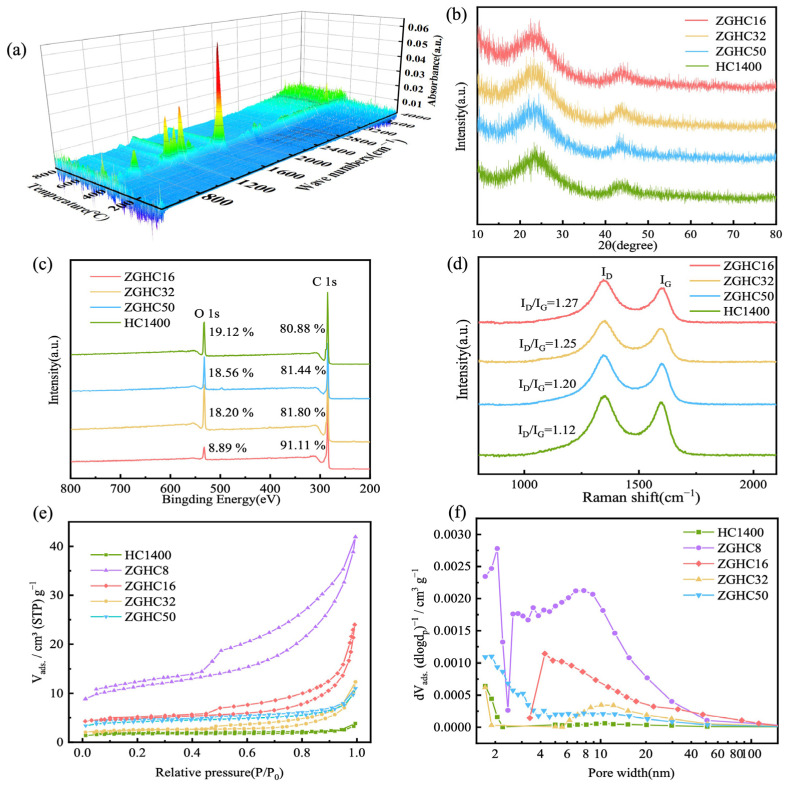
(**a**) Infrared analysis of the pyrolysis products from the mixture of PET and zinc gluconate with a mass ratio of 32:1. (**b**) XRD patterns, (**c**) survey XPS spectra, (**d**) Raman spectra, (**e**) N_2_ adsorption and desorption isotherms, and (**f**) BJH pore-size distribution of HC1400 and ZGHCx.

In order to construct appropriate hard-carbon structures, zinc gluconate was used as pore former, and we studied the co-pyrolysis process of zinc gluconate and PET. Figure 1 illustrates the schematic diagram of the synthesis process for ZGHCx. Figure S1a shows the TGA result for the PET and zinc gluconate mixture (mass ratio of 32:1) from 30 °C to 800 °C, and the corresponding FT-IR analysis for the pyrolysis products is displayed in Figure 2a. The mixtures began to decompose at 432 °C (Figure S1b) and the final carbon yield was 18.21%. During PET pyrolysis, most of the oxygen existed in the gaseous volatiles, which mainly included esters, carbon monoxide, and carbon dioxide. A small amount of oxygen was present in the degradation production with the formation of cross-linking structures [27,30]. Moreover, the organic product mainly consisted of benzene rings, represented by benzoic acid that had escaped during the pyrolysis process [31]. As shown in Figure 2a, massive amounts of C=O, C-O, and C-O-C were detected in the gas products when the heat temperature reached above 432 °C, and this process occurred particularly rapidly, confirming the production of esters, CO, and CO_2_ [32]. XRD was used to further elucidate the phase structure of the obtained carbon samples (Figure 2b). Two peaks around 2θ = 23° and 43° corresponded with the (002) and (100) planes of graphite, respectively. The broad peak width and weak peak intensity confirmed the disordered structure of the hard carbon. The detailed d-interlayer spacing corresponding with the (002) plane could be calculated using the Scherrer Equation (1), as displayed in Table S1. The d-interlayer spacing of all samples was wider than 0.37 nm, and it was thought that this was to allow the sodium ions to be inserted into the carbon layer [17]. The average nanographitic domain length (*La*) and thickness (*Lc*) were calculated using Equation (2).

(1) 2dsinθ=nλ

(2) $$L\left(nm\right)=\frac{k\lambda }{\beta cos\theta }$$

where *λ* is the wavelength (0.15406 nm) of the X-ray and *β* is the full width at half maximum (FWHM) of the peaks. *k* is equal to 1.84 and 0.9 when computing *La* and *Lc*, respectively [28]. Compared with HC1400, ZGHCx had a shorter *La* and *Lc*, which was due to the production of ZnO during the pyrolysis of zinc gluconate. ZnO reacted with PET pyrolytic carbon (C + ZnO = Zn + CO), hindering the growth of the carbon layer and, therefore, tending to produce shorter and thinner graphite domains. Obviously, there were no characteristic peaks of Zn in the final samples. The reason for this was that zinc escaped from the material at high temperatures (vaporization temperature of 907 °C). XPS was used to characterize changes in the carbon and oxygen contents on the surface of the samples. The XPS spectra are presented in Figure 2c. When the proportion of zinc gluconate gradually increased, the oxygen content in the product gradually decreased from 19.12 wt % to 8.89 wt % because the intermediate product of zinc oxide with Lewis acid could promote dehydration and decarboxylation in the reaction process [32]. Figure 2d displays the Raman spectra, showing broad bands at around 1350 cm^−1^ (D band) and 1580 cm^−1^ (G band). The D band and G band corresponded with the disordered graphitic structure and sp^2^ graphitic structure, respectively. As can be seen from Figure S2, the intensity ratio of the D band and G band (I_D_/I_G_) increased with the increase in the proportion of zinc gluconate, suggesting that the degree of graphitization of the samples declined and the degree of surface defects increased. It is generally believed that an increase in surface defects leads to an increase in the sodium storage capacity of the slope region in hard carbon and a decrease in the initial coulombic efficiency (ICE).

The surface area of HC1400 and ZGHCx was tested using the N_2_ adsorption/desorption isotherms, and the results are displayed in Figure 2e. A typical type-II isotherm was obtained for HC1400 and ZGHC50, whereas the isotherms for ZGHC32, ZGHC16, and ZGHC8 appeared to be type IV. The ZnO nanoparticles produced by the pyrolysis of zinc gluconate agglomerated and embedded in the hard carbon. When ZnO reacted with carbon with the formation of Zn, more mesoporous structures were generated, which showed obvious H4 hysteresis in the isotherm. In Figure 2e, it can be seen that the adsorption amounts clearly improved at a relative pressure (P/P_0_) above 0.4 for the samples of ZGHC16 and ZGHC8, indicating their mesoporous structure. The Brunauer–Emmett–Teller (BET) theory was used to calculate the specific surface area (S_BET_), and the BJH method was used to calculate the pore-size distribution. The S_BET_ for HC1400, ZGHC8, ZGHC16, ZGHC32, and ZGHC50 were 6.5 m^2^·g^−1^, 41.3 m^2^·g^−1^, 10.64 m^2^·g^−1^, 9.5 m^2^·g^−1^, and 15.4 m^2^·g^−1^, respectively. Figure 2f shows the pore-size distribution of the samples, confirming the presence of mesoporous structures, especially in ZGHC8 and ZGHC16. There were almost no mesopores in the samples of ZGHC50 and HC1400. The mesopore and macropore volumes of different samples are listed in Table S1. With the addition of zinc gluconate, the volumes for mesopores and macropores obviously increased. This hierarchical structure with mesopores and macropores would help to improve the diffusion of sodium ions in hard carbon while allowing ions to be more easily stored in smaller slit and pore structures.

**Figure 3:**
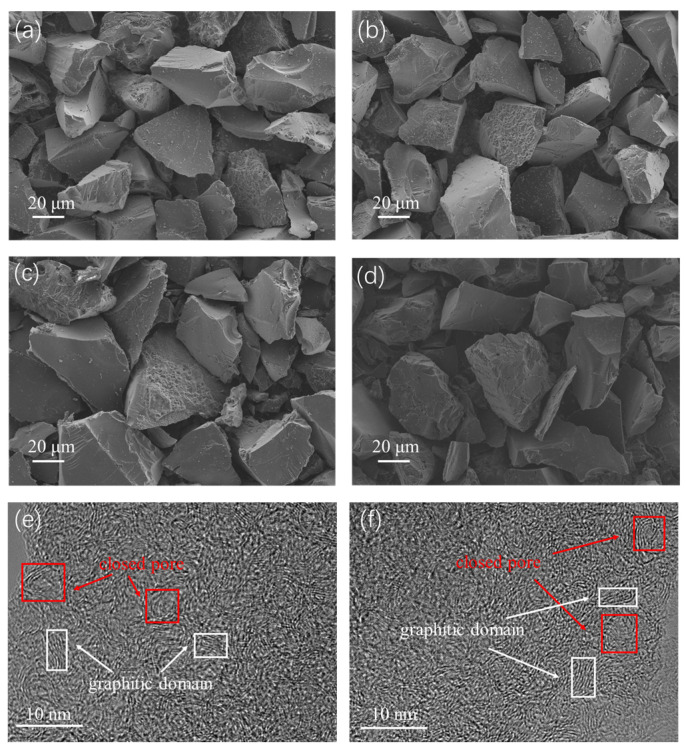
SEM photograph of HC1400 and ZGHCx: (**a**) HC1400; (**b**) ZGHC50; (**c**) ZGHC32; (**d**) ZGHC16. TEM photograph of HC1400 and ZGHC32: (**e**) HC1400; (**f**) ZGHC32. The red box in (**e**,**f**) represents closed pore and the white box means graphitic domain.

As shown in Figure 3a–d, both HC and ZGHCx had irregular microstone-chip morphologies with particle sizes of 30~50 µm, regardless of the additive content of zinc gluconate. The high-resolution SEM images (Figure S3a–d) indicated that the surface of HC1400 was smooth (Figure S3a), but there were visible open pores on the surface of ZGHC16 (Figure S3d), which were caused by the ZnO template. These porous structures allowed the electrolyte to fully penetrate into the material and enhanced the diffusion capacity of the sodium ions. For the microstructure (Figure 3e,f), HC1400 and ZGHC32 exhibited disordered structures composed of short-range nanodomains, curved graphene nanosheets, and closed pores, and these closed pores could provide more sodium-ion storage sites [19,33]. Combined with the results of the N_2_ adsorption/desorption isotherms, it was concluded that zinc gluconate pyrolyzed with the formation of ZnO nanoparticles during the heat treatment, which easily agglomerated into larger ones and became embedded in pyrolytic carbon. When the heat temperature was raised, ZnO was reduced to zinc by carbon. The Zn metal evaporated, leaving a cavity in situ, thus forming mesoporous structures [23,24,34].

### 3.2. Electrochemical Performance Characterization

**Figure 4:**
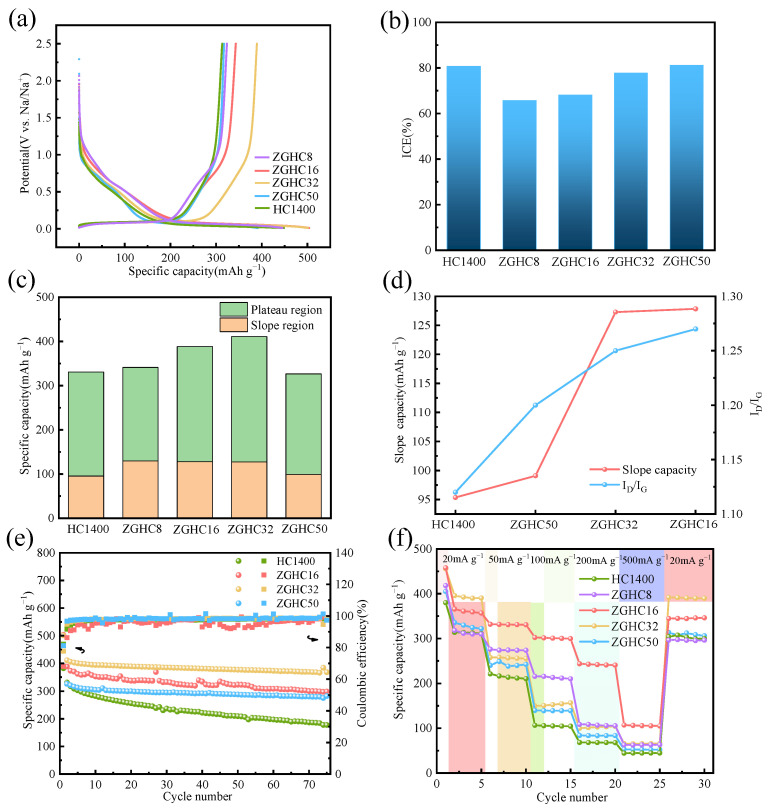
(**a**) The first charge/discharge profiles of HC1400 and ZGHCx. (**b**) The initial coulomb efficiency of the samples. (**c**) The plateau and sloping capacity distribution of different samples. (**d**) Relationship between slope capacity and I_D_/I_G_. (**e**) Long cycle performance and (**f**) rate performance of HC1400 and ZGHCx.

Figure 4a shows the first cycle discharge and charge curves of the HC1400 and ZGHCx samples in the potential range of 0.01~2.5 V at a current density of 20 mA·g^−1^. The discharging capacities of HC1400, ZGHC8, ZGHC16, ZGHC32, and ZGHC50 were 382.32·mAh·g^−1^, 445.25·mAh·g^−1^, 503.70 mAh·g^−1^, 501.02 mAh·g^−1^, and 391.07 mAh·g^−1^, respectively, and the charging capacities were 313.56 mAh·g^−1^, 323.77 mAh·g^−1^, 343.13 mAh·g^−1^, 389.42 mAh·g^−1^, and 317.46 mAh·g^−1^, respectively. The reversible specific capacities of different ZGHC samples were higher than that of HC1400. The reversible capacity of ZG1400, which was prepared by the direct pyrolysis of zinc gluconate at 1400 °C, was only 68.76 mAh·g^−1^, much lower than that of HC1400 (Figure S4). The initial coulomb efficiencies (ICEs) for ZGHC8, ZGHC16, ZGHC32, and ZGHC50 were 65.72%, 68.12%, 77.73%, and 81.18%, respectively (Figure 4b). The ICEs of ZGHCx were a little lower than HC1400 (82.04%). Moreover, with an increase in the additive amount of zinc gluconate, the ICE obviously decreased. The reason for this might be that zinc gluconate built more open-pore structures, which resulted in massive electrolyte decomposition with greater solid electrolyte interphase (SEI) formations. A high specific surface area of 735.6 m^2^·g^−1^ for the sample of ZG1400 was achieved with the formation of more microporous structures, as shown in Figure S5, which led to an extremely low ICE of only 21.69%. Figure 4c shows the storage capacity distribution of the samples, including the sloping capacity (>0.1 V) and the plateau capacity (<0.1 V). The corresponding capacities of HC1400, ZGHC8, ZGHC16, ZGHC32, and ZGHC50 in the slope region were 95.35 mAh·g^−1^, 129.64 mAh·g^−1^, 127.84 mAh·g^−1^, 127.29 mAh·g^−1^, and 99.09 mAh·g^−1^, respectively. The plateau capacities were 235.40 mAh·g^−1^, 224.45 mAh·g^−1^, 260.44 mAh·g^−1^, 283.81 mAh·g^−1^, and 227.3 mAh·g^−1^, respectively. It is generally believed that the capacity of the slope region is related to the adsorption of sodium ions in the defects of hard carbon [17,18,19]. According to the results from the Raman spectroscopy, the I_D_/I_G_ of the hard-carbon samples ranged from 1.12 to 1.27. The capacities of the slope region varied in accordance with these values, indicating that the degree of the defect in the hard carbon was a key factor that contributed to the slope capacity (Figure 4d). In addition, with an increase in the zinc gluconate content, the plateau capacity first increased and then decreased. ZGHC32 showed the highest plateau capacity of 283.81 mAh·g^−1^ at a current density of 20 mA⸱g^−1^. The introduction of zinc gluconate could build mesopores, as concluded from the N_2_ adsorption/desorption isotherms. A proper mesoporous structure was conducive to electrolyte infiltration to form an ion channel so that the sodium ions could be fully stored in the internal unexposed closed-pore structure of the material, resulting in an increased plateau capacity. However, when increasing the amount of zinc gluconate, more mesoporous structures formed at the cost of consuming carbon and destroying the original closed-pore structure, resulting in a significant decrease in the plateau capacity. Obviously, no plateau capacity was observed in the discharge curve of ZG1400. This was because the pyrolysis of zinc gluconate alone would have produced a large number of open micropore structures (Figure S5), thus affecting the capacity of the plateau region. It was also proved that a closed-pore structure was the key factor affecting the capacity of the plateau region.

**Figure 5:**
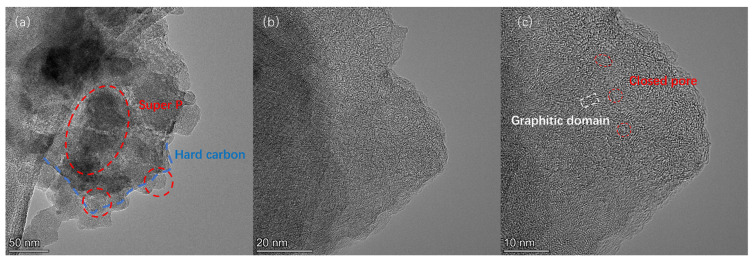
(**a**–**c**) TEM images at different magnifications of ZGHC32 after 75 cycles. The red dashed circle in (**a**) represent Super P and the blue dashed line means hard carbon. The white dashed line in (**c**) means graphitic domain and the red dashed line indicates closed pore.

Figure 4e shows the cycle performance of different samples at 20 mA·g^−1^. Obviously, the cycle stabilities of ZGHCx were much better than that of HC1400. After 75 cycles, the specific capacity of ZGHC32 was 367.73 mAh·g^−1^ and the capacity retention rate was 89.4%, indicating its good cyclic stability. In contrast, the capacity of HC1400 faded to 177.76 mAh·g^−1^ after 75 cycles and the capacity retention rate was only 53.7%. ZGHC16 and ZGHC32 also exhibited capacity retentions of 76.9% and 86.5%, respectively, higher than that of HC1400. The coulombic efficiencies of different samples during the cycling were close to 100%, indicating their high charge utilization ratio. Figure 4f shows the rate capability test results of the different samples. As shown, all HCs suffered from capacity degradation at a high current density. It was interesting to observe that the specific capacity of ZGHC32 was the highest at a current density of 20 mA·g^−1^. When the current density reached 50 mA·g^−1^ and higher, ZGHC16 and ZGHC8 showed better specific capacities. In particular, ZGHC16 exhibited the best performance, with reversible specific capacities of 339.34 mAh·g^−1^, 279.64 mAh·g^−1^, 213.95 mAh·g^−1^, 121.09 mAh·g^−1^, and 53.89 mAh·g^−1^ at current densities of 20 mA·g^−1^, 50 mA·g^−1^, 100 mA·g^−1^, 200 mA·g^−1^, and 500 mA·g^−1^, respectively. The capacities of these ZGHCx samples recovered back to their initial capacities at current densities of 20 mA·g^−1^. The rate of discharge ability reflected the internal dynamics of the anode material, including the conductivity of electrons and the diffusion rate of ions. Abundant ions channels and large carbon-layer spacing were favorable for the enhancement in the ion diffusion rate. The addition of zinc gluconate helped to develop proper mesopores or macropores, which provided a greater transfer path for the electrolyte, benefiting sodium-ion migration [16,35]. Figure 5a–c show the TEM images of ZGHC32 after 75 cycles. It can be seen that the hard carbon was tightly bonded to super-P and had a blocky structure (Figure 5a). In the high-resolution images (Figure 5b,c), the hard carbon after cycling maintained similar structural properties to the initial sample in Figure 3f, which consisted of an obvious graphitic domain and closed-pore structures, indicating its good stability. In addition, Table 1 shows a comparison of this work with some reported studies in terms of precursors, synthesis temperature, and reversible capacity. The present mesoporous hard carbon showed comparable performance to or better performance than the samples obtained using different precursors or synthesis methods at a high current density. 

**Figure 6:**
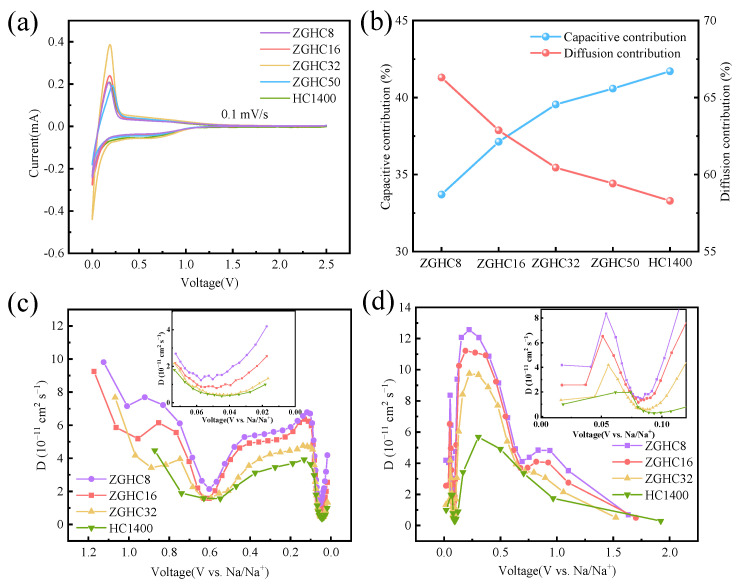
(**a**) CV curves of HC1400 and ZGHCx at a scan rate of 0.1 mV s^−1^. (**b**) Capacitance contribution and diffusion contribution at a scan rate of 0.1 mV s^−1^. (**c**,**d**) Sodium-ion diffusion coefficients of discharge and charge processes for HC1400 and ZGHCx.

The diffusion and pseudo-capacitance behaviors of sodium ions in PET-derived hard carbon were further investigated to study the role of the introduction of zinc gluconate. Figure 6a shows the CV curves of the different samples at 0.1 mV·s^−1^. All samples had pairs of sharp oxidation peaks and reduction peaks at a low potential, which were attributed to the sodiation and desodiation processes in hard carbon, respectively [44,45]. In particular, ZGHC32 exhibited the highest peak current, suggesting its excellent sodium-ion storage capability. Figure S6 shows the CV curves at scanning rates from 0.1 mV·s^−1^ to 2.0 mV·s^−1^. The electrochemical behavior of sodium-ion storage can be characterized using Equation (3).

(3) $$i\left(v\right)={av}^{b}$$

where *i* and *ʋ* are the measured peak current and the scan rate, respectively, and *a* and *b* are the relevant parameters. When the *b* value is 1, it indicates that the sodium-ion storage mechanism of the material is controlled by the capacitance process. The b values calculated from the oxidation peaks of HC1400 and ZGHC50 were 0.64 and 0.63 (Figure S7a,e), indicating the pseudo-capacitive behavior of HC1400 and ZGHC50, and the storage mechanism of the sodium ions was controlled by both diffusion and surface action [27,46,47]. However, the b values for ZGHC8, ZGHC16, and ZGHC32 were 0.49, 0.46, and 0.48, respectively (Figure S7b–d). All of them were close to 0.5, indicating that the sodium storage mechanisms of ZGHC8, ZGHC16, and ZGHC32 were mainly controlled by diffusion. In addition, the degree of contribution of diffusion and capacitance could be quantitatively analyzed using Trasatti’s and Dunn’s Equation (4) [48].

(4) $$i\left(V\right)=k_{1}v+k_{2}v^{1/2}$$

where *i*(*V*) is the peak current and *k*_1_*v* and *k*_2_*v*^1/2^ are capacitance-controlled and diffusion-controlled processes, respectively. Based on the fitting calculation, the capacitance contributions of HC1400, ZGHC8, ZGHC16, ZGHC32, and ZGHC50 at 0.1 mV·s^−1^ were 41.71%, 33.70%, 37.13%, 39.55%, and 40.58% (Figure 6b and Figure S8), respectively. It was found that the capacitance contribution of ZGHC50 was close to that of HC1400. Moreover, with an increase in zinc gluconate, the capacitance contribution decreased from 40.58% for ZGHC50 to 33.7% for ZGHC8. These results indicated that the introduction of zinc gluconate produced more mesopores and macropores, which promoted the diffusion of sodium ions in the materials. Furthermore, we investigated the capacitance contribution at different sweep speeds. The capacitance contribution rates of HC1400 and ZGHCx both increased with an increase in sweep speed, with HC1400 increasing from 41.71% to 91.45% and ZGHC8 increasing from 33.70% to 89.92%. This change trend was due to the increase in the sweep speed; the electrode surface reaction could not take place in time and the storage and release of the charge was borne by the ions in the electrolyte, resulting in an increase in the contribution rate of capacitance [29].

To further explain the diffusion of sodium ions, GITT was used to analyze the diffusion coefficient of sodium ions from different samples during charge and discharge at 20 mA·g^−1^ and at a potential range of 0.01~2.5 V with a pulse time of 30 min and at intervals of 1 h. Based on Fick’s second law, the diffusion coefficient of sodium ions can be calculated using the following equation [49].

(5) $$D_{Na^{+}}=\frac{4}{\pi \tau }{\left(\frac{m_{c}V_{m}}{M_{B}S}\right)}^{2}{\left(\frac{∆E_{s}}{∆E_{\tau }}\right)}^{2}$$

where *τ* is the pulse duration and *m_c_*, *V_m_*, and *M_B_* are the mass of active materials, molar volume, and molecular weight of hard carbon, respectively. *S* is the surface area of the electrodes. Δ*E_s_* and Δ*E_τ_* represent the potential difference between two adjacent open circuits and the voltage shift during a current pulse process, respectively. Figure S9 shows the GITT profiles of HC1400, ZGHC8, ZGHC16, and ZGHC32. The variation in the diffusion coefficient of sodium ions (D_Na_^+^) versus voltage is shown in Figure 6c,d. With an increase in zinc gluconate, the obtained ZGHCx samples exhibited improved D_Na_^+^; ZGHC8 demonstrated the biggest diffusion coefficient among them. During both discharging and charging processes, all samples showed a sharp decrease at 0.1 V in diffusion coefficients, which was attributed to the sodium ions being more easily adsorbed on the surface defects than being inserted into carbon layers and filled with closed pores [50]. The sodiation/desodiation curves could be divided into the following four stages: 1.1~0.6 V, 0.6~0.1 V, 0.1~0.03 V, and 0.03~0.01 V. These might have corresponded with the adsorption of sodium ions on the surface of the material, the filling of the mesopores, the insertion of the carbon layer, and the filling of the closed pores. At a high potential (> 0.6 V), the calculated values of D_Na_^+^ were relatively high, suggesting that the diffusion kinetics of sodium ions under the surface adsorption mechanism were faster than the diffusion-controlled process. Unlike other reports, there was an obvious decline in D_Na_^+^ at a potential of 0.6 V. According to the microstructure of ZGHCx, it was concluded that the filling of mesopores took place here. With the insertion of sodium ions, the sodium-ion diffusivity rose to about 6 × 10^−11^ cm^2^ s^−1^ at a potential of 0.1 V. The sharp decrease in diffusivity from 0.1 V to 0.05 V in the plateau region indicated that intercalation inside the turbostratic domains was more sluggish than the adsorption process on the defect sites [50]. For the process of desodiation, all samples exhibited relatively high diffusion coefficients at the initial desodiation stage when the potential was below 0.01 V, which was due to the pseudo-capacitive behavior of sodium ions within the internal pores [28]. Significantly, a sharp drop in D_Na_^+^ at about 0.03 V could be observed, then D_Na_^+^ rose to a high value of about 10 × 10^−11^ cm^2^ s^−1^, which was attributed to the extraction of sodium ions from closed pores. As the concentration of sodium ions in the electrode decreased, D_Na_^+^ steadily decreased until 0.6 V was reached. There was an obvious fluctuation between 0.6 V and 0.8 V, similar to that in the discharging process, which may have resulted from the extraction of sodium ions from mesopores in the carbon materials. It should be noted that there was no fluctuation for HC1400 in both sodiation and desodiation processes, indicating again the existence of mesopore structures in the ZGHCx samples [51].

Figure S10 demonstrates the EIS measurement results of the different samples. All samples showed typical cell impedance curves, including semicircles in the high-frequency region and oblique straight lines in the low-frequency region related to the charge transfer impedance (R_ct_) and Warburg impedance (Z_w_) of sodium-ion diffusion in the active material, respectively [52]. Compared with HC1400, ZGHCx showed much smaller charge transfer impedances, with R_ct_ values of 673 Ω, 61 Ω, 66 Ω, and 239 Ω for HC1400, ZGHC16, ZGHC32, and ZGHC50, respectively. Z_w_ was also closely related to the amount of zinc gluconate. As the proportion of zinc gluconate increased, the slope of the oblique line in the low-frequency region increased, indicating the better diffusion performance of sodium ions in the hard-carbon materials, which was consistent with the test results of GITT.

## 4. Conclusions

In this work, a series of hard-carbon materials containing mesopores were prepared by pyrolysis of PET and zinc gluconate. The structural parameters of the PET-derived hard carbon and the properties of the prepared hard-carbon negative electrode were demonstrated using material characterization and electrochemical testing. The sample with a mass ratio of PET to zinc gluconate of 32:1 showed the best reversible capacity, producing 389.42 mAh·g^−1^ at a current density of 20 mA·g^−1^. The discharge capacity retention rate was 89.4% after 75 cycles, showing excellent cycle stability. In addition, the introduction of zinc gluconate could help to generate mesopore structures conducive to sodium-ion diffusion, effectively improving the diffusion ability of sodium ions during charge and discharge with a reduction in the charge transfer impedance. This study has proposed a promising approach to converting low-cost PET into high-value carbon-based materials, which could be applied in the fields of energy storage and other renewable energy technology.

## Figures and Tables

**Table 1:** Summary of performance of hard carbon derived from different precursors and methods.

Precursor	Synthesis Temperature (°C)	Reversible Capacity (mAh·g^−1^)	Current Density (mA·g^−1^)	Ref.
Glucose	1300	347.4	30	[36]
3-AP/3-AF resin	1200	360	30	[37]
Rosewood	1100	326	20	[38]
Lignin	1300	328	50	[39]
Corn cob	1300	298	30	[40]
Cotton	1300	315	30	[41]
Kapok	1400	290	30	[42]
PET	900	344	20	[27]
PET	1400	342	20	[28]
PET	1000	337	30	[29]
PET/LC	1200	350	25	[43]
PET	1400	389.42	20	This work
PET	1400	331.93	50	This work

## Data Availability

The original contributions presented in the study are included in the article/Supplementary Materials; further inquiries can be directed to the corresponding author.

## Supplementary Materials

The following supporting information can be downloaded at https://www.mdpi.com/article/10.3390/ma18051166/s1: Figure S1: (a) Thermogravimetric curve of ZGHC32, (b) infrared spectra of pyrolysis products of the mixture of PET and zinc gluconate with a mass ratio of 32:1 at 432 °C; Table S1: Structure information of HC1400 and ZGHCx; Figure S2: I_D_/I_G_ ratio of HC1400 and ZGHCx; Figure S3: SEM photographs of (a) HC1400, (b) ZGHC50, (c) ZGHC32, and (d) ZGHC16; Figure S4: First charge and discharge curves of HC1400 and ZG1400; Figure S5: N_2_ adsorption and desorption isotherms of HC1400 and ZG1400; Figure S6: Cyclic voltammetry curves at different sweep speeds of (a) HC1400, (b) ZGHC8, (c) ZGHC16, (d) ZGHC32, and (e) ZGHC50; Figure S7: Plots of log (sweep rate) versus log (current) of (a) HC1400, (b) ZGHC8, (c) ZGHC16, (d) ZGHC32, and (e) ZGHC50; Figure S8: Capacitive and diffusion contributions at 0.1 mV s^−1^ of (a) HC1400, (b) ZGHC8, (c) ZGHC16, (d) ZGHC32, and (e) ZGHC50; Figure S9: GITT profiles of (a) discharge and (b) charge of HC1400 and ZGHCx; Figure S10: Electrochemical impedance spectroscopy of HC1400 and ZGHCx.
