# Supplementary material for: Tuning the Mesopore Structure of Polyethylene Glycol Terephthalate (PET)-Derived Hard Carbon for High-Capacity Sodium-Ion Batteries
Source: Materials (Basel). 2025 Mar 5;18(5):1166. doi: 10.3390/ma18051166 (PMC11902079; doi:10.3390/ma18051166)
Supplement: Supplementary file 1 [file materials-18-01166-s001.zip › materials-3496641-supplementary.pdf]

Supplementary Material

Tuning Mesopore Structure of Polyethylene Glycol Terephthalate (PET)-Derived Hard Carbon for High-Capacity Sodium-Ion Batteries

Chupeng Wang[1], Mingsheng Luo[1], Shiqi Song[1], Maochong Tang[1], Xiaoxia Wang[2,*], Hui Liu[3]

[1] School of Materials Science and Engineering, East China University of Science and Technology, Shanghai 200237, China

[2] School of Mechanical and Power Engineering, East China University of Science and Technology, Shanghai 200237, China

[3] Zhuguangya Institute of Advanced Science and Technology, Shanghai 201306, China

* Correspondence: wangxiaoxia@ecust.edu.cn

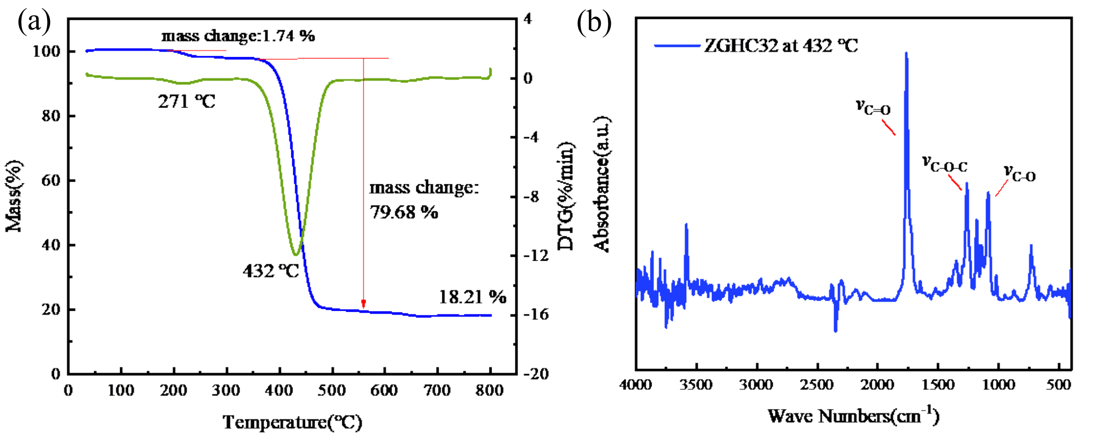

Figure S1. (a) Thermogravimetric curve of the mixture of PET and zinc gluconate with mass ratio of 32:1. (b) Infrared spectra of pyrolysis products of the mixture of PET and zinc gluconate with mass ratio of 32:1 at 432 ℃.

Table S1. Structure information of HC1400 and ZGHCx.

samples	2θ	d_{002} (nm)	L_a (nm)	L_c (nm)	$L_{c/d}$	S_{BET} (m^2/g)	I_D/I_G	$V_{mes.and\ mac.}$ (cm^3/g)
HC1400	23.54	0.378	4.123	2.016	5.333	6.51	1.93	0.0017
ZGHC16	23.84	0.373	3.874	1.895	5.080	41.30	2.42	0.0304
ZGHC32	23.55	0.377	3.865	1.891	5.016	9.49	2.40	0.0137
ZGHC50	23.62	0.376	3.867	1.892	5.032	15.36	2.01	0.0083

Abbreviation: $V_{mes.and\ mac}$: Volume of mesopores and macropores.

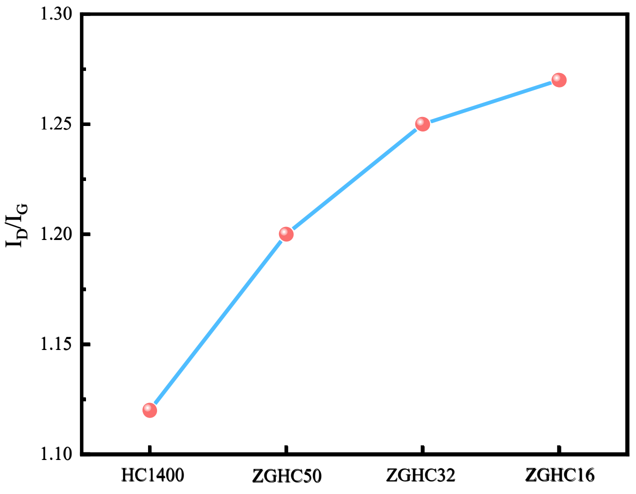

Figure S2. I_D/I_G ratio of the HC1400 and different ZGHCx samples.

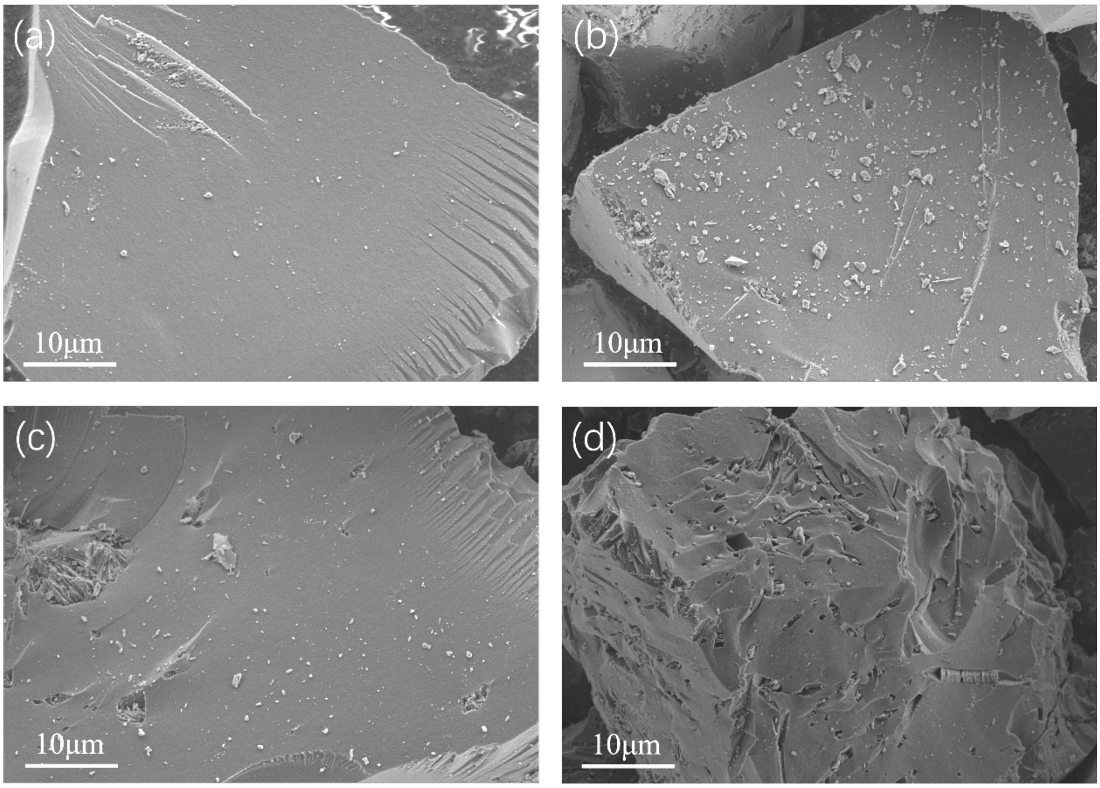

Figure S3. SEM photograph of the HC1400 and ZGHCx: (a) HC1400. (b) ZGHC50. (c) ZGHC32. (d) ZGHC16.

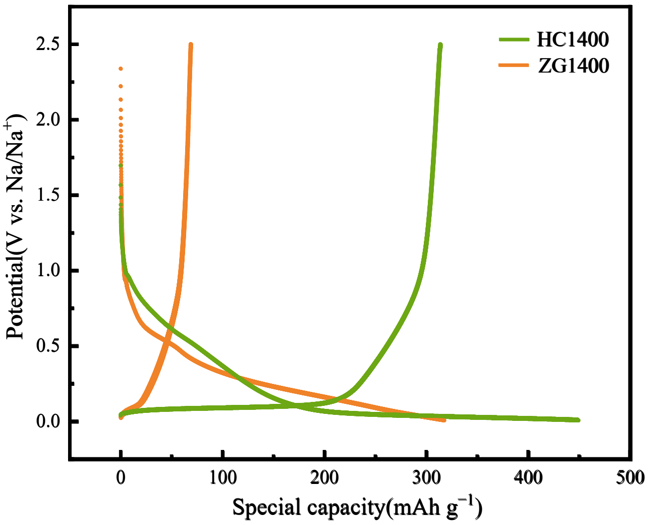

Figure S4. First charge and discharge curves of HC1400 and ZG1400.

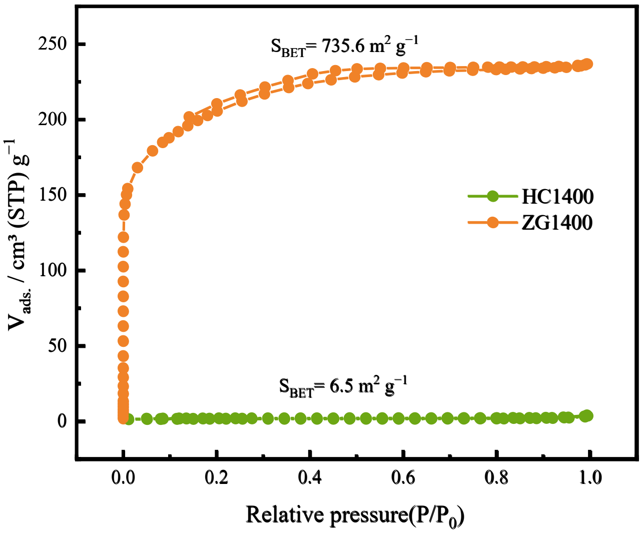

Figure S5. N_2 adsorption and desorption isotherms of HC1400 and ZG1400.

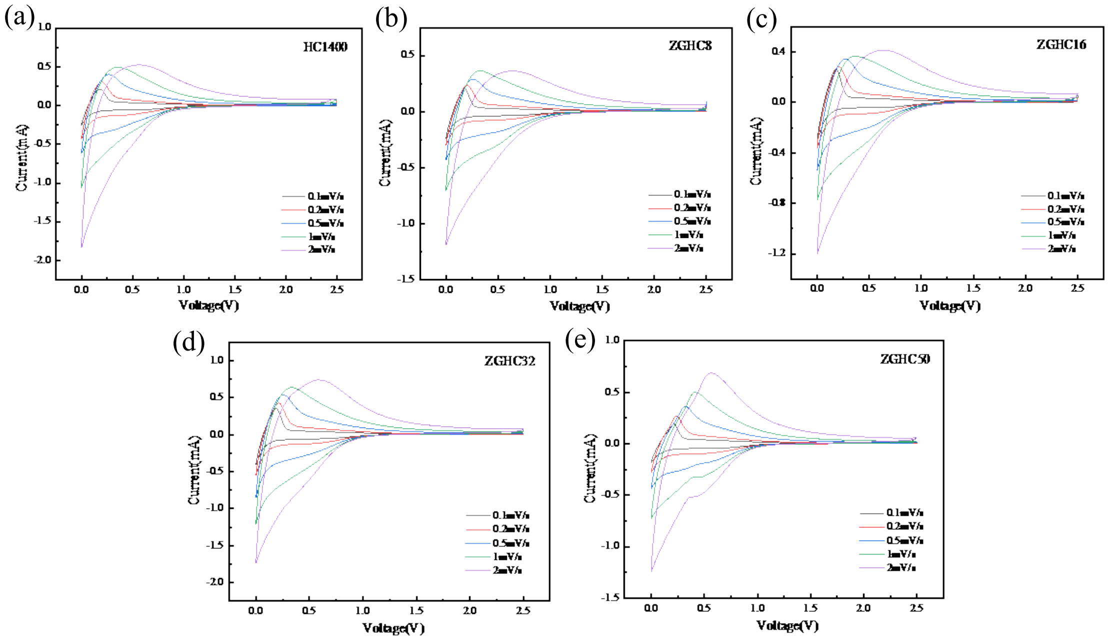

Figure S6. Cyclic voltammetry curves at different sweep speeds of (a) HC1400, (b) ZGHC8, (c) ZGHC16, (d) ZGHC32, and (e) ZGHC50.

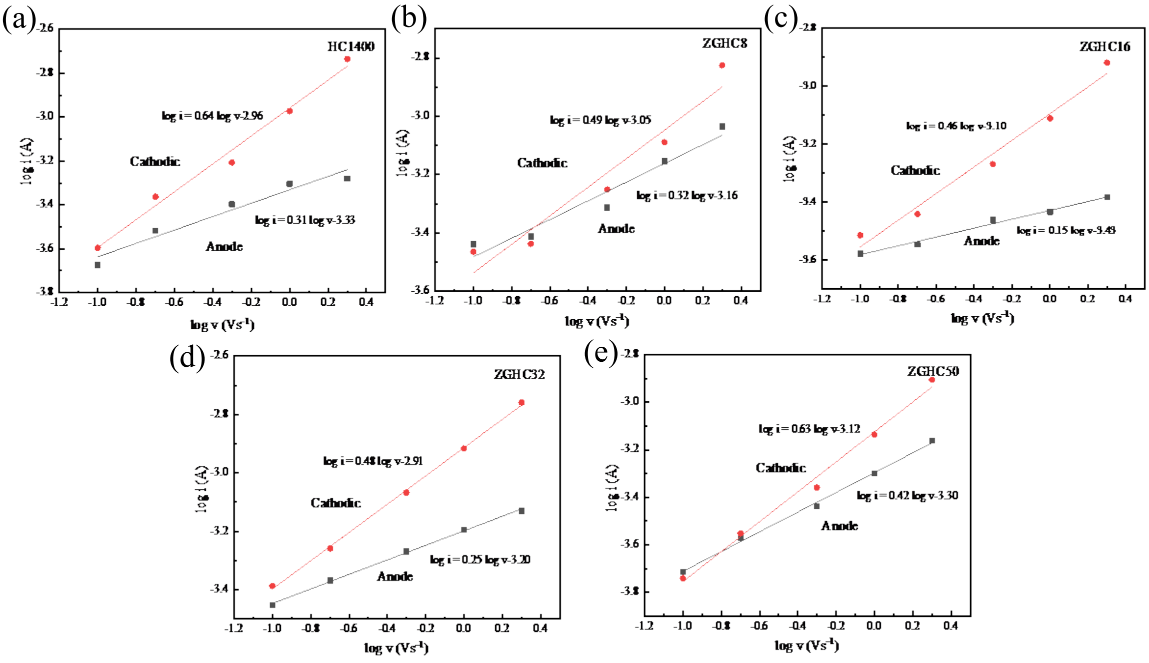

Figure S7. Plots of log (sweep rate) versus log (current) of (a) HC1400, (b) ZGHC8, (c) ZGHC16, (d) ZGHC32, and (e) ZGHC50

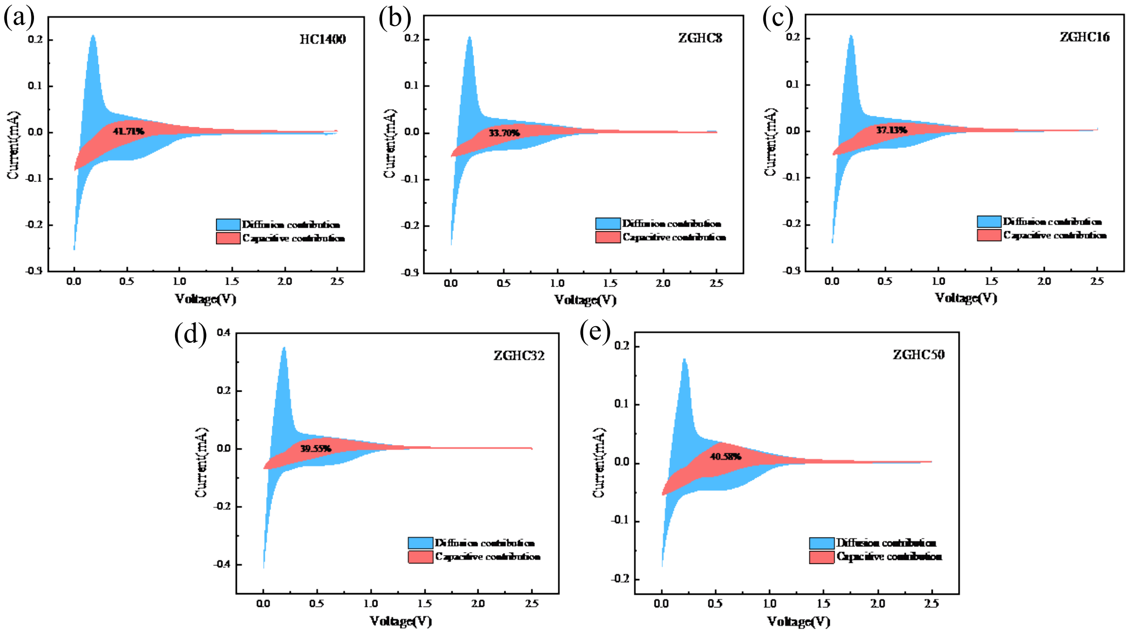

Figure S8. Capacitive and diffusion contributions at 0.1 mV s^{-1} of (a) HC1400, (b) ZGHC8, (c) ZGHC16, (d) ZGHC32, and (e) ZGHC50.

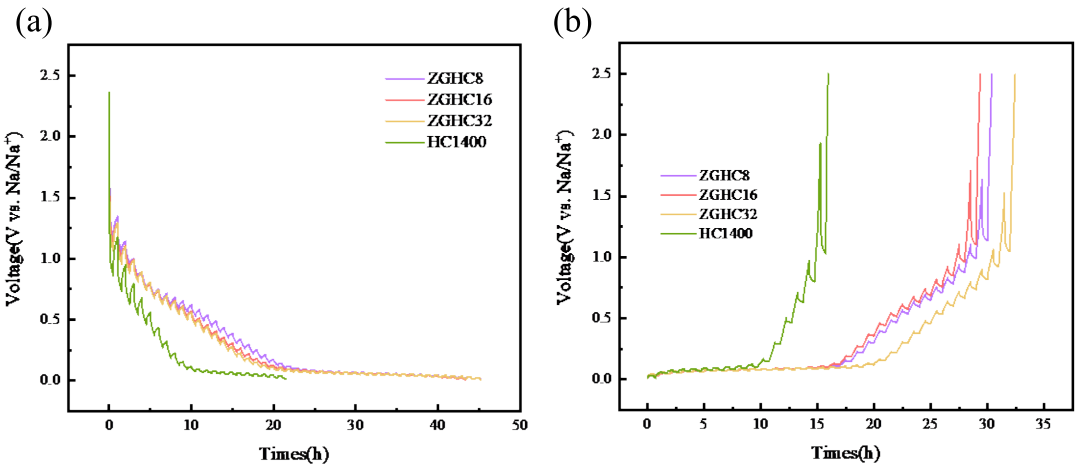

Figure S9. GITT profiles of (a) discharge and (b) charge of HC1400 and ZGHCx.

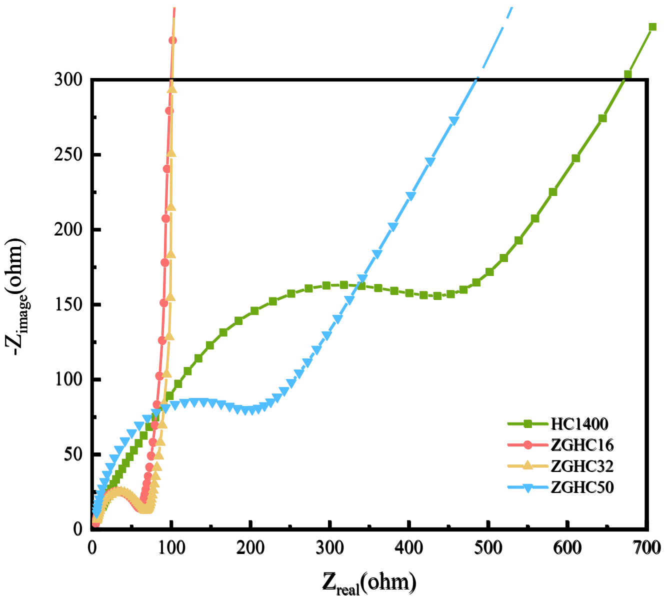

Figure S10. Electrochemical impedance spectroscopy of HC1400 and ZGHCx.
